# Evaluation of Three Methods for CPR Training to Lifeguards: A Randomised Trial Using Traditional Procedures and New Technologies

**DOI:** 10.3390/medicina56110577

**Published:** 2020-10-30

**Authors:** Daniel González-Santano, Daniel Fernández-García, Elena Silvestre-Medina, Beatriz Remuiñán-Rodríguez, Fernando Rosell-Ortiz, Juan Gómez-Salgado, María Sobrido-Prieto, Beatriz Ordás-Campos, Santiago Martínez-Isasi

**Affiliations:** 1 Princesa University Hospital, 28006 Madrid, Spain; daniel_gonzalezsantano@hotmail.com (D.G.-S.); esilvestremedina@gmail.com (E.S.-M.); 2 University Hospital of Leon, 24071 León, Spain; dfernandezg@saludcastillayleon.es (D.F.-G.); beaordass@gmail.com (B.O.-C.); 3 University Hospital Complex A Coruña, Galician Health Service (SERGAS), University of A Coruña, 15006 A Coruña, Spain; bearemuinan@gmail.com; 4 La Rioja 061 Emergency and Urgent Care Department, 26007 Logroño, Spain; fernandorosell@gmail.com; 5 Department of Sociology, Social Work and Public Health, Faculty of Labour Sciences, University of Huelva, 21007 Huelva, Spain; 6 Safety and Health Postgraduate Programme, Universidad Espíritu Santo, Guayaquil 092301, Ecuador; 7 Department of Health Sciences, School of Nursing and Podiatry, University of A Coruña, 15006 A Coruña, Spain; mordasaria.sobrido@udc.es; 8 CLINURSID Research Group, Santiago de Compostela’s Health Research Institute (IDIS), Faculty of Nursing, University of Santiago de Compostela, 15705 Santiago de Compostela, Galicia, Spain; smtzisasi@gmail.com

**Keywords:** cardiopulmonary resuscitation, education, teaching, mobile application, applied health technology, health technology products

## Abstract

*Background and objectives:* When the drowning timeline evolves and drowning occurs, the lifeguard tries to mitigate the event by applying the last link of the drowning survival chain with the aim of treating hypoxia. Quality CPR (Cardiopulmonary Resuscitation) and the training of lifeguards are the fundamental axes of drowning survival. Mobile applications and other feedback methods have emerged as strong methods for the learning and training of basic CPR in the last years so, in this study, a randomised clinical trial has been carried out to compare the traditional method as the use of apps or manikins with a feedback system as a method of training to improve the quality of resuscitation. *Materials and Methods:* The traditional training (TT), mobile phone applications (AP) and feedback manikins (FT) are compared. The three cohorts were subsequently evaluated through a manikin providing feedback, and a data report on the quality of the manoeuvres was obtained. *Results:* Significant differences were found between the traditional manikin and the manikin with real-time feedback regarding the percentage of compressions with correct depth (30.8% (30.4) vs. 68.2% (32.6); *p* = 0.042). Hand positioning, percentage correct chest recoil and quality of compressions exceeded 70% of correct performance in all groups with better percentages in the FT (TT vs. FT; *p* < 0.05). *Conclusions:* As a conclusion, feedback manikins are better learning tools than traditional models and apps as regards training chest compression. Ventilation values are low in all groups, but improve with the feedback manikin.

## 1. Introduction

Drowning is a public health problem, and according to the World Health Organization, “every day and every hour, more than 40 people lose their lives to drowning in the world” [1]. Epidemiology is different depending on the country, although there are variables that seem to influence the number of deaths by drowning such as age, sex, and the location of the event [2]; drowning is common in inland waters and during summer months [3].

Drowning timeline sorts and reflects the triggers, actions, and interventions associated to the drowning process [4]. At the time the person must be rescued, the timeline has failed and event mitigation is required through the application of the last link cover, “provide care as needed”, of the drowning survival chain [3,5]. This link reflects the basic life support (BLS) sequence in drowning, where treating hypoxia is critical [3].

Since oxygen deficiency is the main cause of death by drowning, oxygenation and ventilation are the priority actions in drowning situations, either in the aquatic environment or out of it. Resuscitation begins with ventilation; mouth-to-mouth is more effective and rapid, but it is better using a bag-valve-mask, which is the most reasonable option when performed by 2 rescuers [2], as well as 100% oxygen administration [2,3].

The lifeguard should know the physiopathology of drowning and the particularities of the drowned patient so as to provide the best care. Among the possible caring actions, quality CPR (Cardiopulmonary Resuscitation) with chest compressions and effective ventilations is essential, being a determining factor for the survival of drowned victims [2,3]. In addition, another determining element is effective training [6].

In Spain, lifeguards have seasonal work and they have other occupations the rest of the year. This particular situation results in a need for training at the beginning of the summer, as skills decrease at 3–6 months after training [7].

In recent years, different training and self-directed education methods have been developed, being alternatives to the training of health and lay staff [6]. Some of the new trends in CPR training are mobile applications (apps) that provide support during the early stages of the survival chain and/or during CPR compressions training by using the ability to detect and calculate the compression depth and the frequency of compressions per minute in real time. The main advantage of this tool is that it is a more universal, easy-to-manage and cost-saving method [8].

In recent years, manikins have been developed with feedback capability. They mainly report CPR skills: Compression frequency, depth, chest recoil, and hands positioning, being an important alternative in the training of non-experts and also healthcare professionals [6,9,10].

The objective of our study was to compare the traditional method with the use of apps or manikins with feedback system as training methods to improve the quality of resuscitation.

## 2. Materials and Methods

The study was designed as a non-blinded randomised trial, involving a convenience sample of 30 beach lifeguards during the months of June to September 2018.

### 2.1. Pre-Phase

**Figure 1:**
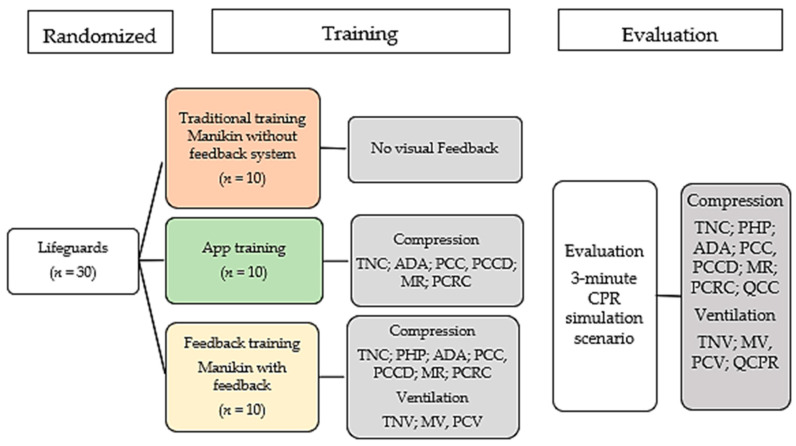
Flow chart of the study design. ADA: mm of depth achieved; MR: Mean rate; MV: Mean volume; PCC: % compressions at correct depth; PCV: % correct ventilations (PCV); PCCD: % correct chest recoil; PCRC: % correct rate compressions; PHP: % hands positioning; QCC: Quality of compressions; QCPR: Overall quality of CPR; TNC: Total number of compressions; TNV: Total number of ventilations.

The sample was randomised by distributing the 30 lifeguards into 3 groups with 3 different training methods: Traditional training (TT), who received training with a manikin without feedback system guided by the instructor; app training (AP), who received training using an app (*Massage cardiaque et DSA*) and, finally, feedback training (FT), who received training with a manikin with feedback (Figure 1). The lifeguards received the same training; a 12-min session of training and practised at least 6 min of CPR. The maximum lifeguard/teacher and lifeguard/manikin ratio was 4/1. The teacher was a nurse with training in emergency situations and teaching experience in BLS. All lifeguards had been trained in BLS in the previous 2 years.

### 2.2. Test Phase

Between 7 and 15 days after the training, each lifeguard performed an evaluation of a 3-min CPR simulation scenario. In the study, the figure of 70% was used as overall CPR quality, which some experts have appointed as a cut-off point for sufficient quality [11].

### 2.3. Instruments

The instrument used in the training were the app, the Resusci Anne QCPR Skillreporter and Resusci Anne manikin without feedback system (Figure 1).

The app was Massage cardiaque et DSA designed by IMAOS SAS, free and downloaded on a smartphone. The app provided the following parameters related to the quality of compressions as total number of compressions, mm of depth achieved, % compressions at correct depth, % correct chest recoil, mean rate, and % correct rate compressions.

The Resusci Anne QCPR Skillreporter (Laerdal, Norway) provided total number of compressions, % hands positioning, mm of depth achieved, % compressions at correct depth, % correct chest recoil, mean rate, % correct rate compressions, total number of ventilations, mean volume, % correct ventilations, and QCPR.

The final evaluation was conducted by the Resusci Anne QCPR Skillreporter (Laerdal, Norway).

### 2.4. Variables

The dependent variables of the study were those derived from the Resusci Anne QCPR Skillreporter and calculated the QCC (quality of compressions) score using the equation [12,13]:

[(% compressions at correct depth + % correct chest recoil + % correct rate compressions)/3].

### 2.5. Ramdomisation

It was done by randomisation of lifeguards, carried out through the Excel^®^ software for Windows 10 (Microsoft, Redmond, WA, USA) RAND function.

### 2.6. Statistical Analysis

For the study of the quantitative variables, normality was tested using the Shapiro–Wilk test. The quantitative variables were expressed by central trend and dispersion measures (mean (standard deviation or SD); median (interquartile range)) according to normality. The qualitative variables were expressed by using absolute and relative frequencies. The mean comparison was done by the Mann–Whitney U test, and the multiple comparison of means was performed through the ANOVA test with Bonferroni correction (assuming equal variances) or the Games-Howell test (not assuming equal variances). To assess the variances homogeneity, the Levene ANOVA test with Bonferroni correction was used for those variables with normal distribution, and the Kruskall–Wallis test for those that did not meet this assumption. The data was processed and analysed using the SPSS v.21.0 statistical package (IBM, Armonk, NY, USA). A significance level of *p* < 0.05 was established.

### 2.7. Ethical Considerations

All lifeguards obtained previous information on the purpose and procedure of this study and stated their desire to voluntarily participate through the informed consent. The participation in the study did not imply any risk or benefit for the participants. Data were anonymously collected and recorded, maintaining at all times the confidentiality of the information. The study was conducted in accordance with the Declaration of Helsinki, and the protocol was approved by the Ethics Committee for Research of the University of León (ETICA-ULE-022-2018) on 20 July 2018.

## 3. Results

Thirty lifeguards participated with a mean age of 26.9 (SD: 7.1), and 86.67% (26/30) of them were men. No difference was found between the groups regarding age (*p* = 0.179) or sex (*p* = 0.315).

### 3.1. Compressions

**Figure 2:**
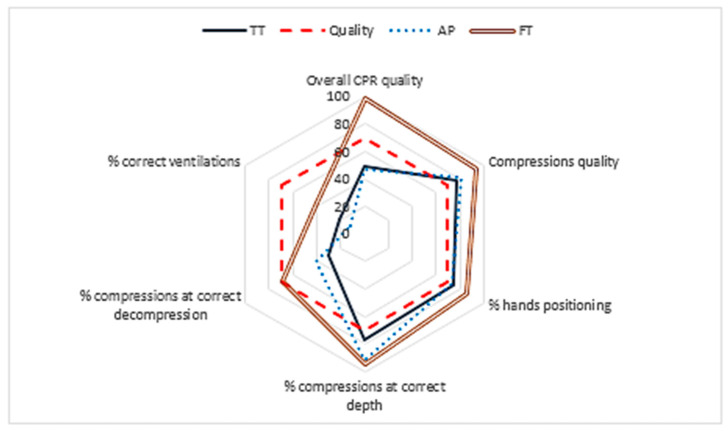
Percentages of CPR quality variables for each group. TT: Traditional training; AP: App training; FT: feedback training.

With regard to the mean depth variable, it was observed that the TT did not reach the 50 mm recommended by the ERC (European Resuscitation Council), with no difference found between the groups. The compression percentage with the correct depth showed differences between the groups (TT vs. AP vs. FT) (*p* = 0.047), being significant between the TT and the FT (*p* = 0.047) (Table 1 and Figure 2).

The mean rate of chest compressions/minute in the TT was higher than the one recommended by the ERC, and 75% of lifeguards had a higher rate than recommended (Table 1).

As for the quality of compressions (QCC) (Table 1), significant differences were observed, with FT obtaining better results, as compared to the TT and AP (*p* = 0.039), the difference between FT and TT was significant (*p* = 0.045).

The variables that exceeded 70% were % hands positioning, percentage of complete chest recoil and quality of compressions in all groups.

### 3.2. Ventilations

The FT achieved better results than the TT, with significant differences in the total number of ventilations (*p* = 0.043) (Table 1 and Figure 2). The mean insufflation volume was higher than the one recommended by the ERC in the TT and AP (Table 1) although no significant differences were observed between groups.

### 3.3. CPR Quality

Differences were observed in the overall quality of CPR (QCPR), with a higher percentage in the FT than in the TT and AP (*p* = 0.027). Significant differences were found between FT and AP (*p* = 0.014) and between FT and TT (*p* = 0.028) (Table 1).

## 4. Discussion

This study has shown how feedback manikin training improves results in parameters that determine the quality of compressions and ventilations, as compared to other training methods.

Apps are readily available and easy-to-use resources that are not regulated, as most of them cannot guarantee their validity and reliability [14]. Fernandez et al. [8] studied 3 different apps for CPR training without obtaining differences, but did find improvements in the rate of chest compressions per minute. Our study used one of the apps assessed in this mentioned study.

The decision to accommodate the duration of CPR manoeuvres to this time period is based on the quality loss of the compressions after 2 min of resuscitation, as described in a study that covered the effect that physical fatigue had on lifeguards when applying the former four minutes, the authors observed differences regarding non-fatigued lifeguards [15].

The measured parameters obtained a quality level similar to the one found in previous studies [6,15] that used similar training methods as regards the variables: Correct hands positioning, correct chest recoil, and rate of compression per minute.

The app achieved a different percentage of compressions with correct depth (no significant differences between AP vs. TT and AP vs. FT), as compared to the standard manikin and the feedback manikin, as in previous studies [16].

Significant differences were only found between the manikin with feedback as compared to the other 2 groups regarding the overall quality of CPR because, although without finding significant differences, the results are better with the feedback manikin as regards the variables that determine the quality of CPR.

Other CPR quality parameters that showed improvement without statistical differences were the mean volume and the percentage of adequate ventilations, and both were higher in the manikin with feedback as it provided real-time information. However, the data from our study do not match those by Zapletal et al. [16], where training with an app or a standard manikin obtained the mean volume within the suggested range (500–600 mL). Also, in our study, the percentage of adequate ventilations was very low in the three groups. This must be highlighted due to the importance of lifeguards performing appropriate ventilations at cardiac arrest situations where treating hypoxia is fundamental. Therefore, and from what has been observed in other studies, ventilation training with feedback can be an appropriate resource to improve the quality of ventilations.

Anyway, the practise of ventilation is currently unsafe and should be avoided, although resuscitation attempts will be considered if safety conditions exist and if the lifeguard team has specific training and PPE. This is because with the advent of Covid-19, there are extra complications during resuscitation due to the aerosol generation during compressions and the need to perform ventilations safely, using barrier materials, antiviral filters and personal protection [17].

In our study, we have compared feedback manikin training and standard manikin training. This goes in line with the study by Baldi et al. [18], where better results and significant differences were obtained in the percentage of compressions with the right depth when training with feedback manikin as compared to the standard manikin, just like in our study.

The availability of manikins with real-time feedback has improved the quality of training, as compared to other types of training in CPR skills [16]. Other tools such as mobile apps may be helpful in training parameters such as rate of compressions per minute and in training with limited resources.

Our study presents several limitations. On the one hand, some lifeguards had already received previous training, which was minimised with practical training prior to evaluation given by the same supervisor. On the other hand, the sample size which, despite being small, included a group of lifeguards with a very high degree of motivation. Also, the assessment was done in a simulated context which does not exactly represent a real CPR situation, so this may be a limitation when extrapolating results to real possible victims. Effectivity of ventilations was not assessed; instead, ventilations in which an airflow was produced were measured, allowing the person in training to try these but in a non-effective way. It is possible that by increasing the time and/or modifying the training method, the obtained results may be better. Other relevant limitations may be motivational factors, stress, etc., which can only be evaluated in real events.

Finally, it is highlighted as a limitation of the study that this intervention has not been able to be performed again after a reasonable period of time of 2 weeks, after which the retention of abilities usually begins to disappear. So that, a new intervention is proposed as a future line of investigation to compare the retention capacity in all three groups after a while since training.

## 5. Conclusions

It can be concluded that feedback manikins are better learning tools for chest compression training, as compared to manikins without feedback and the app, regarding parameters that determine the quality of compressions, such as the mean depth.

Ventilation values are low in all groups and improve in the feedback manikin, but without reaching the agreed quality value (70%). In this case, further studies focusing on the feedback about ventilation training may be needed.

## Figures and Tables

**Table 1:** Quality of chest compressions elements and ventilations after training.

Variables		TT (A)	AP (B)	FT (C)	Inter-Group *p*	Intra-Group *p*
Total number of compressions	*Media (SD)*	254.1 (54.8)	253.9 (35.6)	247.2 (47.9)	0.932	
	*Median (IQ)*	252.5 (233.0–304.0)	251.0 (231.0–263.0)	240.5 (209.0–284.0)		
% hands positioning	*Mean (SD)*	74.8 (26.7)	73.0 (36.2)	85.7 (28.5)	0.154	
	*Median (IQ)*	80.7 (60.9–99.4)	89.5 (63.6–98.2)	99.8 (96.7–100.0)		
Millimetres of depth achieved	*Mean (SD)*	47.6 (8.8)	50.3 (5.8)	54.1 (4.9)	0.112	
	*Median (IQ)*	47.0 (40.0–56.0)	49.0 (46.0–56.0)	55.5 (51.0–58.0)		
% compressions at correct depth	*Mean (SD)*	30.8 (30.4)	40.5 (34.2)	68.2 (32.6)	0.042	A vs. C = 0.047
	*Median (IQ)*	24.5 (0.0–49.0)	34.0 (15.0–61.0)	75.5 (51.0–96.0)		
% correct chest recoil	*Mean (SD)*	76.8 (26.4)	91.7 (13.3)	94.6 (9.9)	0.139	
	*Median (IQ)*	86.1 (56.3–99.6)	99.8 (76.6–100.0)	99.5 (95.0–100.0)		
Mean rate	*Mean (SD)*	125.5 (22.6)	112.9 (11.5)	118.5 (15.5)	0.276	
	*Median (IQ)*	131.0 (120.0–143.0)	112.0 (108.0–121.0)	114.0 (112.0–130.0)		
Quality of compressions	*Mean (SD)*	77.7 (16.2)	81.7 (10.9)	93.8 (13.9)	0.039	A vs. C = 0.045
	*Median (IQ)*	73.6 (67.8–92.2)	82.2 (74.1–85.3)	95.1 (89.9–104.0)		
Total number of ventilations	*Mean (SD)*	15.7 (3.9)	15.3 (1.4)	14.2 (1.8)	0.048 *	A vs. C = 0.043 **
	*Median (IQ)*	16.5 (16.0–18.0)	16.0 (16.0–16.0)	14.0 (14.0–16.0)		
Mean volume	*Mean (SD)*	788.0 (281.7)	762.2 (274.8)	541.1 (115.7)	0.069	
	*Median (IQ)*	755.0 (630.0–950.0)	700.0 (670.0–870.0)	560.0 (480.0–580.0)		
% correct ventilations	*Mean (SD)*	21.6 (29.6)	11.6 (18.0)	41.1 (33.1)	0.087	
	*Median (IQ)*	8.5 (0.0–31.3)	6.3 (0.0–12.5)	42.9 (14.3–75.0)		
Overall quality of CPR	*Mean (SD)*	49.4 (17.3)	46.5 (10.6)	68.0 (18.6)	0.027 *	A vs. C = 0.028 ** B vs. C = 0.014 **
	*Median (IQ)*	41.4 (37.9–56.4)	48.4 (38.3–53.0)	69.0 (51.9–73.5)		

TT: standard manikin; AP: APP Massage cardiaque et DSA; FT: feedback manikin. Standard deviation: SD; Interquartile range: IQ. Non-parametric tests: Kruskall–Wallis * and Mann–Whitney **.

## References

[1] World Health Organization (2014). Global Report on Drowning.

[2] Abelairas-Gómez C., Tipton M.J., González-Salvado V., Bierens J.J.L.M. (2019). El ahogamiento: Epidemiología, prevención, fisiopatología, reanimación de la víctima ahogada y tratamiento hospitalario. Emergencias.

[3] Truhlář A., Deakin C.D., Soar J., Khalifa G.E.A., Alfonzo A., Bierens J.J., Brattebø G., Brugger H., Dunning J., Hunyadi-Antičević S. (2015). European Resuscitation Council Guidelines for Resuscitation 2015: Section 4. Cardiac arrest in special circumstances. Resuscitation.

[4] Szpilman D., Tipton M., Sempsrott J., Webber J., Bierens J., Dawes P., Seabra R., Barcala-Furelos R., Queiroga A.C. (2016). Drowning timeline: A new systematic model of the drowning process. Am. J. Emerg. Med..

[5] Szpilman D., Webber J., Quan L., Bierens J.J., Morizot-Leite L., Langendorfer S.J., Beerman S., Løfgren B. (2014). Creating a drowning chain of survival. Resuscitation.

[6] Greif R.R., Lockey A.S., Conaghan P.P., Lippert A.A., De Vries W.W., Monsieurs K.G., Ballance J.H.W.J., Barelli A., Biarent D., Bossaert L. (2015). European Resuscitation Council Guidelines for Resuscitation 2015: Section 10. Education and implementation of resuscitation. Resuscitation.

[7] Monsieurs K.G., Nolan J.P., Bossaert L.L., Greif R., Maconochie I.K., Nikolaou N.I., Perkins G.D., Soar J., Truhlář A., Wyllie J. (2015). European Resuscitation Council Guidelines for Resuscitation 2015: Section 1. Executive summary. Resuscitation.

[8] Fernández-Méndez F., Barcala-Furelos R., Otero-Agra M., Santos-Folgar M., Rodríguez-Núñez A., Fernández-Méndez M. (2020). Evaluation of the thoracic compression technique using APPs. Do they help or hinder cardiopulmonary resuscitation?. Med. Intensiv..

[9] Ringh M., Rosenqvist M., Hollenberg J., Jonsson M., Fredman D., Nordberg P., Järnbert-Pettersson H., Hasselqvist-Ax I., Riva G., Svensson L. (2015). Mobile-phone dispatch of laypersons for CPR in out-of-hospital cardiac arrest. N. Engl. J. Med..

[10] Yeung J., Meeks R., Edelson D., Gao F., Soar J., Perkins G.D. (2009). The use of CPR feedback/prompt devices during training and CPR performance: A systematic review. Resuscitation.

[11] Perkins G.D., Colquhoun M., Simons R., Colquhoun M., Handley A.J., Evans T.R. (2004). Training manikins. ABC of Resuscitation.

[12] Méndez-Martínez C., Martínez-Isasi S., García-Suárez M., De La Peña-Rodríguez M.A., Gómez-Salgado J., Fernández D. (2019). Acquisition of Knowledge and Practical Skills after a Brief Course of BLS-AED in First-Year Students in Nursing and Physiotherapy at a Spanish University. Int. J. Environ. Res. Public Health.

[13] Fungueiriño-Suárez R., Barcala-Furelos R., González-Fermoso M., Martínez-Isasi S., Fernández-Méndez F., González-Salvado V., Navarro-Patón R., Rodríguez-Núñez A. (2018). Coastal Fishermen as Lifesavers While Sailing at High Speed: A Crossover Study. BioMed. Res. Int..

[14] Martín-Fernández A., Marco-Cuenca G., Salvador-Oliván J.A. (2020). Evaluación y acreditación de las aplicaciones móviles relacionadas con la salud. Rev. Esp. Salud Pública.

[15] Abelairas C., Romo V., Barcala R.J., Palacios J. (2013). Efecto de la fatiga física del socorrista en los primeros cuatro minutos de la reanimación cardiopulmonar posrescate acuático. Emergencias.

[16] Zapletal B., Greif R., Stumpf D., Nierscher F.J., Frantal S., Haugk M., Ruetzler K., Schlimp C., Fischer H. (2014). Comparing three CPR feedback devices and standard BLS in a single rescuer scenario: A randomised simulation study. Resuscitation.

[17] Barcala-Furelos R., Aranda-García S., Abelairas-Gómez C., Martínez-Isasi S., López-Mesa F., Oleagordia-Aguirre A., Palacios-Aguilar J., Szpilman D. (2020). Recomendaciones de salud laboral para socorristas ante emergencias acuáticas en la era Covid-19: Prevención, rescate y reanimación. Rev. Esp. Salud Pública.

[18] Baldi E., Cornara S., Contri E., Epis F., Fina D., Zelaschi B., Dossena C., Fichtner F., Tonani M., Di Maggio M. (2017). Real-time visual feedback during training improves laypersons’ CPR quality: A randomized controlled manikin study. CJEM.

